# Different Expression and Clinical Implications of Cancer-Associated Fibroblast (CAF) Markers in Brain Metastases

**DOI:** 10.7150/jca.80115

**Published:** 2023-02-05

**Authors:** Md Rashedunnabi Akanda, Eun-Jung Ahn, Yeong Jin Kim, S M Abdus Salam, Myung-Giun Noh, Sung Sun Kim, Tae-Young Jung, In-Young Kim, Chang-Hyun Kim, Kyung-Hwa Lee, Kyung-Sub Moon

**Affiliations:** 1 Department of Pathology, Chonnam National University Research Institute of Medical Science, Chonnam National University Hwasun Hospital and Medical School, Hwasun, Jeollanam-do, South Korea.; 2 Department of Neurosurgery, Chonnam National University Research Institute of Medical Science, Chonnam National University Hwasun Hospital and Medical School, Hwasun, Jeollanam-do, South Korea.; 3 Department of Surgery, Chonnam National University Research Institute of Medical Science, Chonnam National University Hwasun Hospital and Medical School, Hwasun, Jeollanam-do, South Korea.; 4 Department of Pharmacology and Toxicology, Sylhet Agricultural University, Sylhet, Bangladesh.; 5 BioMedical Sciences Graduate Program (BMSGP), Chonnam National University, Hwasun Jeollanam-do, South Korea.

**Keywords:** Alpha-smooth muscle actin, Brain metastasis, Cancer-associated fibroblasts, Platelet-derived growth factor receptor-beta, Tumor microenvironment

## Abstract

**Aims:** This study assessed the expression and clinical relevance of cancer-asssociated fibroblast (CAF)-related biomarkers in brain metastasis (BM). Moreover, molecular characterization of patient-derived primary CAFs and normal fibroblasts (NFs) was performed.

**Methods:** Sixty-eight patients with BM from various primary cancer types were selected. Immunohistochemistry (IHC) and immunofluorescence (IF) staining were performed to evaluate the expression of various CAF-related biomarkers. CAFs and NFs were isolated from fresh tissues.

**Results:** Various CAF-related biomarkers were expressed in CAFs in BMs of different primary cancers. However, only PDGFR-β, α-SMA, and collagen type I were associated with BM size. PDGFR-β and α-SMA were associated with BM recurrence after resection. PDGFR-β was associated with recurrence-free survival (RFS). Interestingly, high expression of PDGFR-β and α-SMA was found in the patients with previous chemotherapy or radiotherapy for primary cancer. In primary cell culture, PDGFR-β and α-SMA were expressed at higher levels in patient-derived CAFs than in NFs or cancer cells. The origins of CAF in BM were presumed to be pericytes of blood vessels, circulating endothelial progenitor cells, or transformed astrocytes of the peritumoral glial stroma.

**Conclusion:** Our results suggest that high expression of CAF-related biomarkers, particularly PDGFR-β and α-SMA, is associated with poor prognosis and recurrence in patients with BM. With the elucidation of the role and origins of CAF in the tumor microenvironment, CAF can be a new imperative target for BM immunotherapy.

## Introduction

Brain metastases (BMs) are the most frequently diagnosed intracranial neoplasms in adults and are associated with poor prognosis and high mortality. Approximately 20% of patients with lung, skin, kidney, breast, colon, and hematological malignancies progress to BM 1. Patients diagnosed with BM are commonly treated with a combination of surgery, chemo-radiotherapy, and targeted therapies. BM is associated with poor prognosis, with a median survival of less than 6 months 2. Moreover, the development of resistance to conventional treatment in patients with BM is not uncommon, resulting from individual patient variation and minimal blood-brain barrier penetration of chemotherapy 3.

Stromal cells in the tumor microenvironment (TME) play crucial roles in metastasis, allowing for migration, colonization, and growth of tumor cells 4. TME consists of extracellular matrix (ECM) and various cell types, including cancer cells, stromal cells, fibroblasts, cancer-associated fibroblasts (CAFs), mesenchymal stem cells (MSCs), immune cells, endothelial cells, and pericytes 5. CAFs are activated fibroblasts and comprise the principal stromal components in several malignancies 6. CAF activation is regulated by the reciprocal signaling between tumor cells and CAFs through a variety of cytokines, chemokines, and growth factors. Accumulating evidence suggests an important role of CAFs in enhancing tumor progression and metastasis by promoting ECM remodeling, cancer cell proliferation, angiogenesis, epithelial-to-mesenchymal transition (EMT), and endothelial-to-mesenchymal transition (EndMT), creating a niche that permits the development of drug-resistance 7, 8. In the TME, stromal CAFs can develop from various types of cells, such as normal fibroblasts (NFs), epithelial cells through EMT, endothelial cells through EndMT, bone marrow-derived cells (BMDCs), adipocytes, and stellate cells 9. The relationship between CAF, tumor status and patient prognosis remains controversial in various human tumors 10, 11. Therefore, to target the tumor-promoting CAF subsets, it is crucial to identify markers specifically expressed in these cell populations. Although different CAF subsets can be distinguished by their CAF gene expression profiles 12, no specific surface markers for CAF subsets exist, limiting their relevance in the development of effective targeted therapies for patients with BM.

Activated CAFs exhibit elevated expression of several surface biomarkers that distinguish them from NFs. The expression of such biomarkers is associated with poor prognosis and tumor recurrence. These CAF-related markers include alpha-smooth muscle actin (α-SMA) 12-14, fibroblast activation protein alpha (FAP-α) 15, fibroblast specific protein 1 (S100A4/FSP1) 16, platelet-derived growth factor receptor (PDGFR-β) 17, collagen type I 18, nerve/glial antigen 2 (NG2) 19, Tenascin-C 20 and Twist1 21. Therefore, CAFs can be classified into different functional subtypes, according to the expression of these markers 22. However, the detailed molecular characterization of the different CAF subtypes and their clinical relevance in BM remain largely unknown. In the present study, we quantified and characterized stromal CAFs in human BM based on the expression of CAF-related biomarkers using immunohistochemistry (IHC), as well as investigated their clinical relevance. The sources of stromal CAFs were determined based on IHC and immunofluorescence (IF) findings. We also isolated primary CAFs and NFs from BM and skin, respectively, and characterized their features using IF and Western blotting (WB) analyses. We identified PDGFR-β and α-SMA as important CAF-related biomarkers in BM and concluded that CAFs could be key players in the progression of BM, representing an imperative target for BM immunotherapies.

## Materials & Methods

### Human tissue specimens and clinical data

Our study included 68 BM samples from patients with different primary cancer types. BM specimens and clinical information were obtained from pathology archives and clinical records of patients who underwent craniotomy and tumor removal at the Chonnam National University Hwasun Hospital between 2004 and 2018. Of 382 surgical cases of metastatic carcinoma that were submitted from the neurosurgery department, 315 cases were first screened by intracranial location and tissue availability. After the primary screening of intracranial location and paraffin block condition, a relatively large number of cases remained on the list for the lung, breast, colon, and liver cancer, but relatively few cases for gastric cancer (11 cases), melanoma (9 cases), kidney cancer (8 cases), and thyroid (3 cases). In order to study the presence and distribution of BM CAFs from various primary cancers, we decided to select around 10 cases by organ after reviewing the list. It was judged that it could reflect the differences in various primary cancer organs and the general characteristics of BM. Among organs with a small number of remaining cases, gastric cancer, melanoma, and kidney cancer were included without additional screening, and thyroid was excluded due to a small number. In cases of the lung, breast, colon, and liver cancer, which have a relatively large number of BM cases, 10 cases of each were selected with a priority of the existence of matched primary cancer tissues and the order of recent cases. The presence or absence of other diseases of the patients was not considered in case selection. The case numbers of primary organ cancers at each selection step and detailed diagnoses of primary cancer were summarized in Table S1.

The following data were obtained retrospectively from the medical records: age, sex, tumor size, presence of other metastases, presence of chemotherapy or radiation therapy for primary cancer prior to BM, Karnofsky performance score, symptoms, synchronous or metachronous detection of BM, the number of BM, tumor location, resection range (gross total resection or not), and adjuvant radiation therapy. Recurrence-free survival (RFS) was calculated from the date of surgery to the date of local recurrence (new lesion on operation site in the cases with gross total resection, or progression in the cases with non-gross total resection). Of 68 cases, 61 patients underwent serial enhanced MRI scans to detect a local recurrence at 1-3 months' interval. Written informed consent was obtained from patients or their legal surrogates for the use of clinical information and pathological specimens. The study was approved by the Institutional Review Board of Chonnam National University Hwasun Hospital (CNUHH-2019-218).

### Immunohistochemical staining and interpretation

Hematoxylin and eosin (H&E) stained sections of BM were examined, and representative tissue blocks were selected for further staining. Tissue sections (3 μm thick) were subjected to IHC using a Bond-max autostainer (Leica Microsystems, Buffalo Grove, IL, USA), as previously described 23. The following antibodies were used: α-SMA (1:100 dilution; catalogue no. ab5694; Abcam, Cambridge, UK), FAP-α (1:1000 dilution; catalogue no. AF3715; R&D, Minneapolis, USA), S100A4/FSP1 (1:1000 dilution; catalogue no. HPA007973; Atlas, Bromma, Sweden), PDGFR-α (1:100 dilution; catalogue no. sc398206; Santa Cruz, Texas, USA), PDGFR-β (1:400 dilution; catalogue no. ab32570; Abcam), collagen type I (1:300 dilution; catalogue no. ab34710, Abcam), NG2 (1:600 dilution; catalogue no. ab139406; Abcam), Tenascin-C (1: 50 dilution; catalogue no. sc25328; Santa Cruz), and Twist1 (1:100 dilution; catalogue no. ab50887; Abcam). IHC slides were assessed by experienced pathologists (SSK and KHL), who were blinded to the clinical details. The staining intensity in stromal CAFs was initially graded from 0 through 3 and then grouped into two categories. The scores of immunostaining in stromal CAFs was given according to the relative ratio of the area stained by the CAF markers to the area of the tumor cells: 0, no staining; 1, ≤ 3%; 2, ≤ 10%; and 3 > 10%. Low expression was graded with 0 or 1, while high expression was graded with 2 or 3 for α-SMA, FAP-α, S100A4/FSP1, PDGFR-β, collagen type I, NG2, and Tenascin-C. Twist1 expression was defined as positive/high when nuclear staining was present, and as negative/low when nuclear staining was absent.

### Cell culture and conditioned media preparation

The cell lines LLC1 (mouse lung cancer), MDA-MB-231 (human breast cancer), A549 (human lung cancer), and MC-38 (mouse colon cancer) were purchased from the American Type Culture Collection (ATCC; Manassas, VA, USA). GL261 (mouse glioma) was kindly provided by Dr. Maciej S. Lesniak (Northwestern University Feinberg School of Medicine, Chicago, IL, USA). These cancer cell lines were used as negative controls in the expectation that they would not express CAF markers. LLC1, A549, MC-38, and GL261 were maintained in Dulbecco's Modified Eagle Medium (DMEM; Thermo Scientific, Waltham, MA, USA), while MDA-MB-231 was maintained in Roswell Park Memorial Institute Medium (RPMI; Thermo Scientific, Waltham, MA, USA). The cell culture medium was supplemented with 10% fetal bovine serum (FBS; GIBCO, Invitrogen, Carlsbad, CA, USA), as well as 100 U/mL penicillin and 100 μg/mL streptomycin (Sigma-Aldrich, St. Louis, MO, USA). All cell lines were cultured in a 37°C incubator with a humidified atmosphere of 5% CO_2_. For conditioned media preparation, human cancer cell lines (A549 and MDA-MB-231) were grown to confluency (70-80%), followed by a wash with phosphate-buffered saline (PBS) and replacement of the growth media with DMEM. The cells were cultured for an additional 24 h, and the medium was collected and centrifuged for 10 min at 12000 rpm. Then the conditioned media were passed through a 0.22 µm filter, and stored in aliquots at -80°C until further use. Cell culture media and conditioned media were used at a 2:1 ratio in subsequent experiments.

### Establishment of primary CAFs and NFs

Fresh human tissue from BM and normal human skin tissue specimens were obtained from patients who underwent surgery. Primary CAFs and NFs from BMs were established using a previously described method with minor modifications 21. Briefly, fresh tissues were obtained immediately after surgery and were cut into small pieces (≤1 mm). Tissue samples were enzymatically dissociated in DMEM/F12 containing collagenase I (1 mg/mL, Sigma-Aldrich), DNase I (0.1 mg/mL, STEMCELL Technologies, Vancouver, Canada), and normocin (100µg/mL, InvivoGen, San Diego, CA, USA) at 37°C for 1 h using a shaker. To separate stromal fibroblasts, samples were centrifuged at 700 rpm for 5 min, and cells were collected from the supernatant by centrifugation at 800 rpm for another 8 min. After washing with PBS, fibroblasts were cultured in DMEM/F12 media supplemented with 10% FBS and 1% penicillin-streptomycin. All CAFs and NFs used in the experiments were at early passages (≤3).

### Western blotting analyses

Proteins were extracted from lysed cells using radioimmunoprecipitation assay (RIPA) buffer (Bio Solution, Seoul, South Korea) containing protease inhibitor cocktail (Roche, Mannheim, Germany). Equal amounts of protein (30 μg) were separated by 8-10% polyacrylamide gel electrophoresis (PAGE) containing 0.1% sodium dodecyl sulfate (SDS) and electrophoretically transferred onto polyvinylidene fluoride membranes (GE Healthcare, Marlborough, MA, USA). The membranes were incubated with blocking buffer (5% skim milk) at room temperature (RT) for 1 h, followed by probing for 16 h at 4°C with primary antibodies: α-SMA (1:1000, ab7817, Abcam), FAP-α (1:1000, ab53066, Abcam), PDGFR-β (1:5000, ab32570, Abcam), pan-Cytokeratin (pan-CK, 1:1000, SC 8018, Santa Cruz), and β-actin (1:5000, 3700, Cell Signaling, Danvers, USA). Subsequently, membranes were incubated for 1 h at RT with anti-rabbit or anti-mouse immunoglobulin secondary antibodies conjugated to horseradish peroxidase (HRP). Protein bands were detected using an electrochemiluminescence system (Millipore, Burlington, MA, USA), and signal intensities were quantified using an ultraviolet (UV) imaging system (LAS 4000 Image Quant system; GE Healthcare). β-actin was used as a loading control.

### Immunofluorescence staining

IF staining of CAFs and NFs was performed as previously described 24. Cultured cells were fixed with 4% formaldehyde, followed by dehydration using a series of alcohol solutions. After blocking with 2% bovine serum albumin (BSA) for 30 min at RT, cells were permeabilized with 0.1% Triton X-100 for 20 min. Then they were incubated for 16 h at 4°C with primary antibodies targeting α-SMA (1:200, ab7817, Abcam) and PDGFR-β (1:100, ab32570, Abcam). Subsequently, samples were co-incubated with goat anti-mouse IgG (1:400, A-11001, Life Technologies, Carlsbad, CA, USA) and goat anti-rabbit IgG (1:400, A1011, Life Technologies) secondary antibodies for 1 h at RT.

In addition, formalin-fixed paraffin-embedded (FFPE) human BM tissues were cut into sections 3 mm thick and deparaffinized using xylene. Heat-induced antigen retrieval was performed in citrate buffer (pH=6.0) for 15 min, and 3% hydrogen peroxide was used for the inactivation of endogenous peroxidase activity. Then the tissues were incubated for 16 h at 4°C with primary antibodies: α-SMA (1:200, ab7817, Abcam), α-SMA (1:100, ab5694, Abcam), PDGFR-β (1:100, ab32570, Abcam), CD34 (1:50, M7165, Dako), and GFAP (1:200, 3670, Cell Signaling Technology). Subsequently, tissues were co-incubated with goat anti-mouse IgG (1:400, A-11001, Life Technologies) and goat anti-rabbit IgG (1:400, A1011, Life Technologies) secondary antibodies for 1 h at RT. Nuclei were counterstained with 300 nM DAPI for 20 min, followed by a wash with PBS. The chamber slides were mounted with antifade mounting media and imaged using the EVOS fluorescence imaging system (Thermo Fisher Scientific, Waltham, MA, USA).

### Statistical analyses

All data were analyzed using SPSS version 23.0 software for Windows (SPSS, Chicago, IL, USA) and Graph Pad Prism version 6.00 (Graph Pad, La Jolla, CA, USA). Comparisons in tumor size, as well as the presence of local recurrence and clinicopathological variables, were analyzed with the chi-square test, and binary logistic regression was applied for univariate and multivariate analyses. The effects of different clinicopathological variables on RFS were determined using univariate Kaplan-Meier method with log-rank tests. *P*-values < 0.05 were considered statistically significant.

## Results

### Expression of stromal CAF-related biomarkers in BM

**Fig 1:**
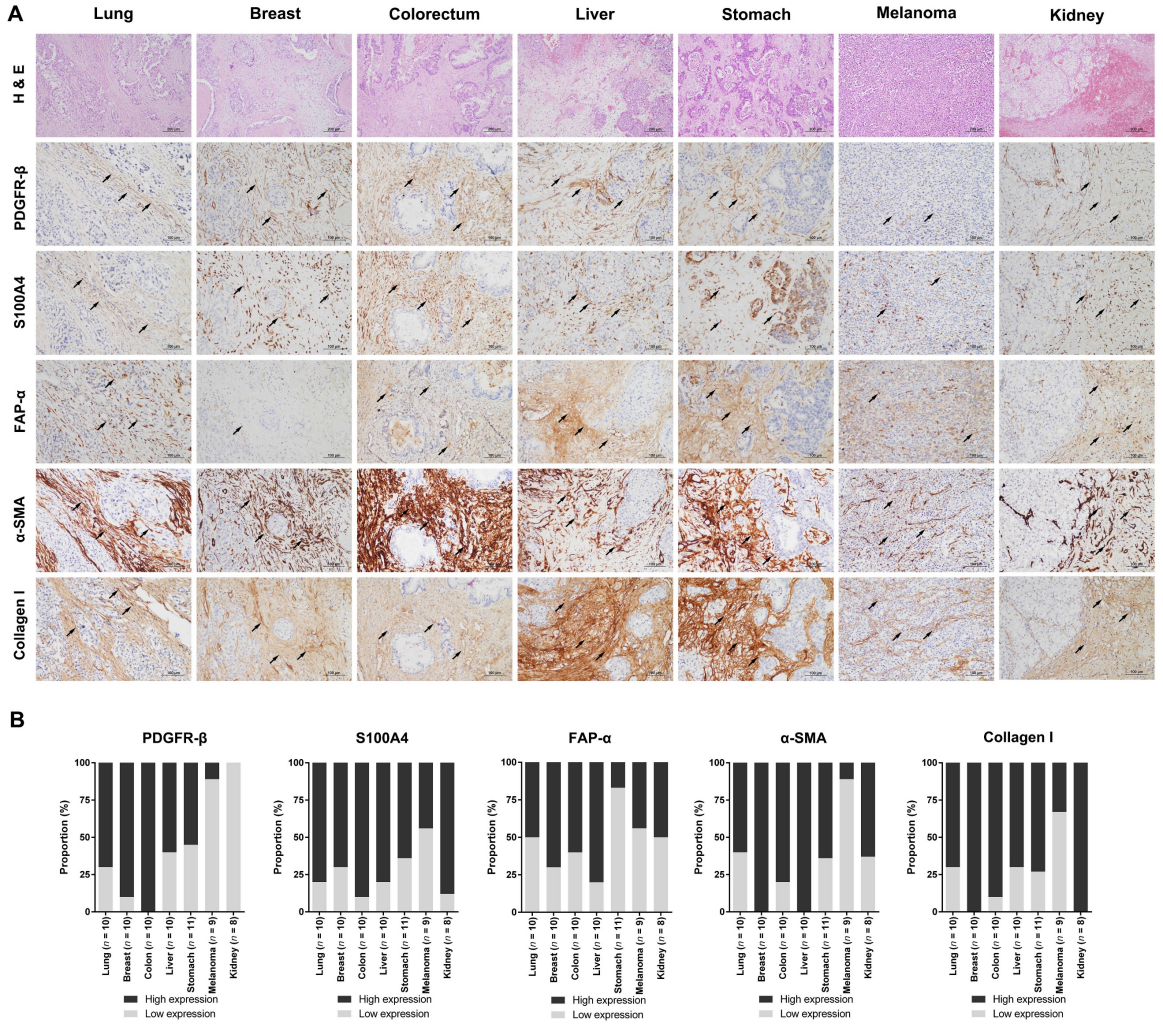
** Microphotographs displaying the expression of CAF-related biomarkers in BMs from different primary cancer types.** (a) The expression of CAF-related markers, including PDGFR-β, S100A4/FSP1, FAP-α, α-SMA, and collagen type I, was prominent in stromal CAFs (black arrowheads) whereas the parenchymal tumor cells were negative for these markers (H&E stains, original magnification ×100, top row; IHC, original magnification ×200, except the top row). (b) The expression of CAF-related biomarkers was high in a significant proportion of patients with lung, breast, colon, liver, and stomach cancer. BMs from melanoma had relatively low levels of CAFs, as indicated by the low rate of cases with high expression of CAF-related biomarkers. Histology images were taken from the same BM case for each primary cancer organ.

**Fig 2:**
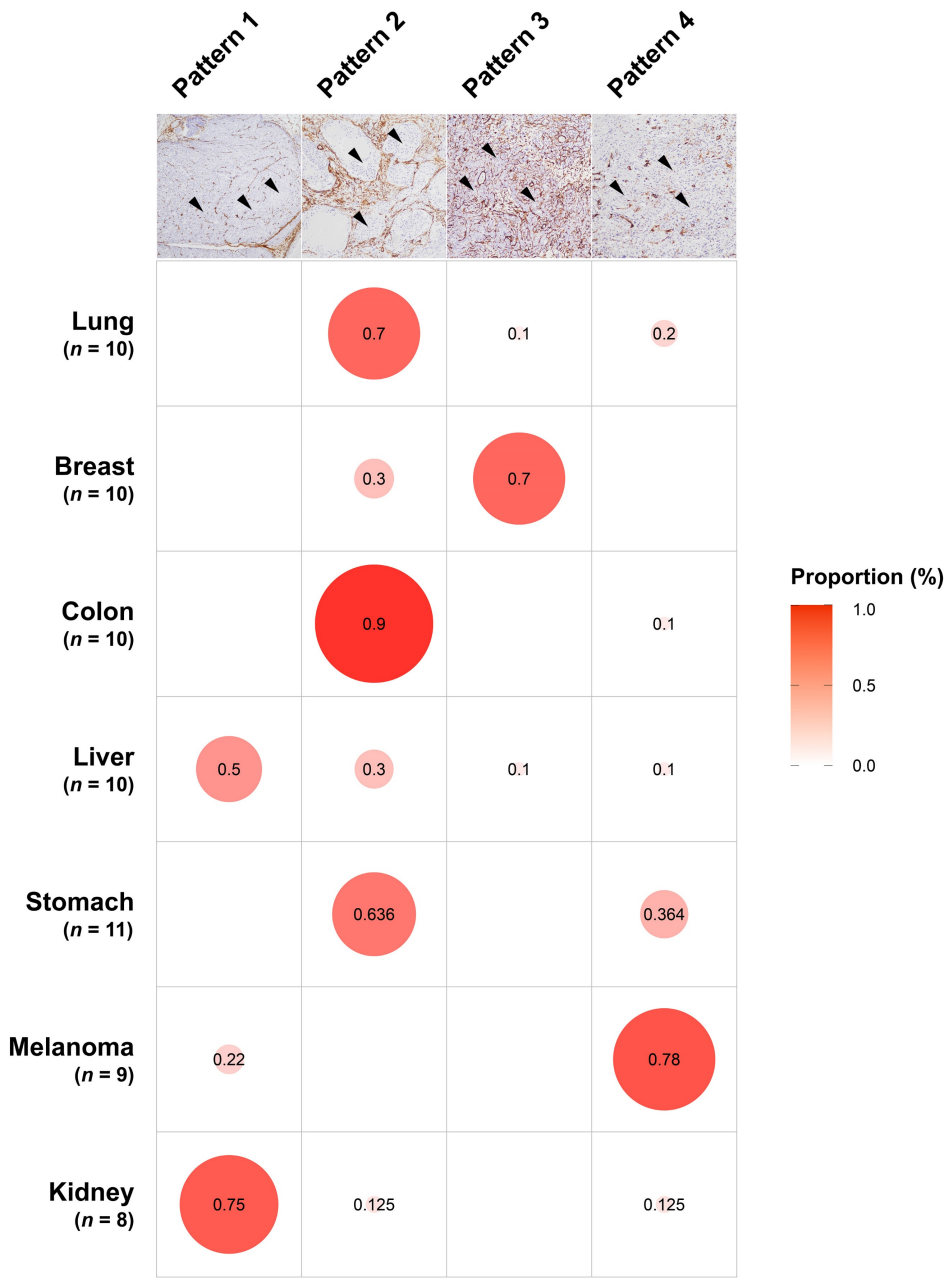
** The patterns of CAF distribution in association with primary cancer types.** In pattern 1, the CAFs were distributed in the periphery of the large tumor nests (black arrowheads), mainly seen in BMs from kidney and liver cancers. In pattern 2, CAFs were located around medium-to-large tumor cell clusters (arrowheads), typically observed in BMs from the lung, colorectum, and stomach cancers. The CAFs in pattern 3 encased individual cancer cells or intermingled closely with small tumor nests (arrowheads), prominently in BMs from breast cancers. In pattern 4, the CAFs were scarcely present (arrowheads in tumor cells) and this pattern was most predominant in metastatic melanoma. The size of the circles was drawn by reflecting the frequency of the pattern in each primary cancer type.

To investigate the levels of stromal CAFs in BMs, we performed IHC analyses using BM tissues from 68 patients with different primary cancer types, including lung, breast, colorectum, liver, stomach, melanoma, and kidney. The expression of various CAF-related biomarkers was assessed to characterize the stromal CAFs in BMs. A distinct expression pattern of PDGFR-β, S100A4/FSP1, FAP-α, α-SMA, and collagen type I in the stromal CAFs was observed in relation to the tumor area (Fig. 1). These five biomarkers showed prominent expression in the surrounding and intervening tumor stroma areas. In contrast to the tumor stroma, the expression of these CAF-related biomarkers in the tumor area was almost entirely negative. Twist1 was also highly expressed in the nuclei of stromal CAFs in tissues from patients with primary cancer in lungs, breast, colorectal, and liver, whereas NG2 (primary tumors in breast, colorectal, liver, and kidney) and Tenascin-C (primary tumors in lungs and breast) were expressed only in a small number of cases. Although we could not detect PDGFR-α expression in stromal CAFs, its expression was notably high in the tumor region (Fig. S1). The distribution of CAFs varied significantly according to the primary cancer type and could be grouped into four patterns (Fig. 2). In pattern 1, the CAFs were typically located in the outer rim of the expansile tumor nests, mainly seen in BMs from kidney and liver cancers. In pattern 2, CAFs were primarily distributed around medium-to-large tumor cell clusters and this pattern was observed mainly in BMs from the lung, colorectum, and stomach cancers. The CAFs in pattern 3 most often encased individual cancer cells or intermingled closely with small tumor nests, most prominently in BMs from breast cancers. The CAFs in metastatic melanoma were relatively scarce compared to BMs from other primary cancer types and this was categorized as pattern 4. Taken together, the expression intensity and distribution pattern of BM CAFs markers varied according to the primary cancer organ.

### Origins of CAF in BM

**Fig 3:**
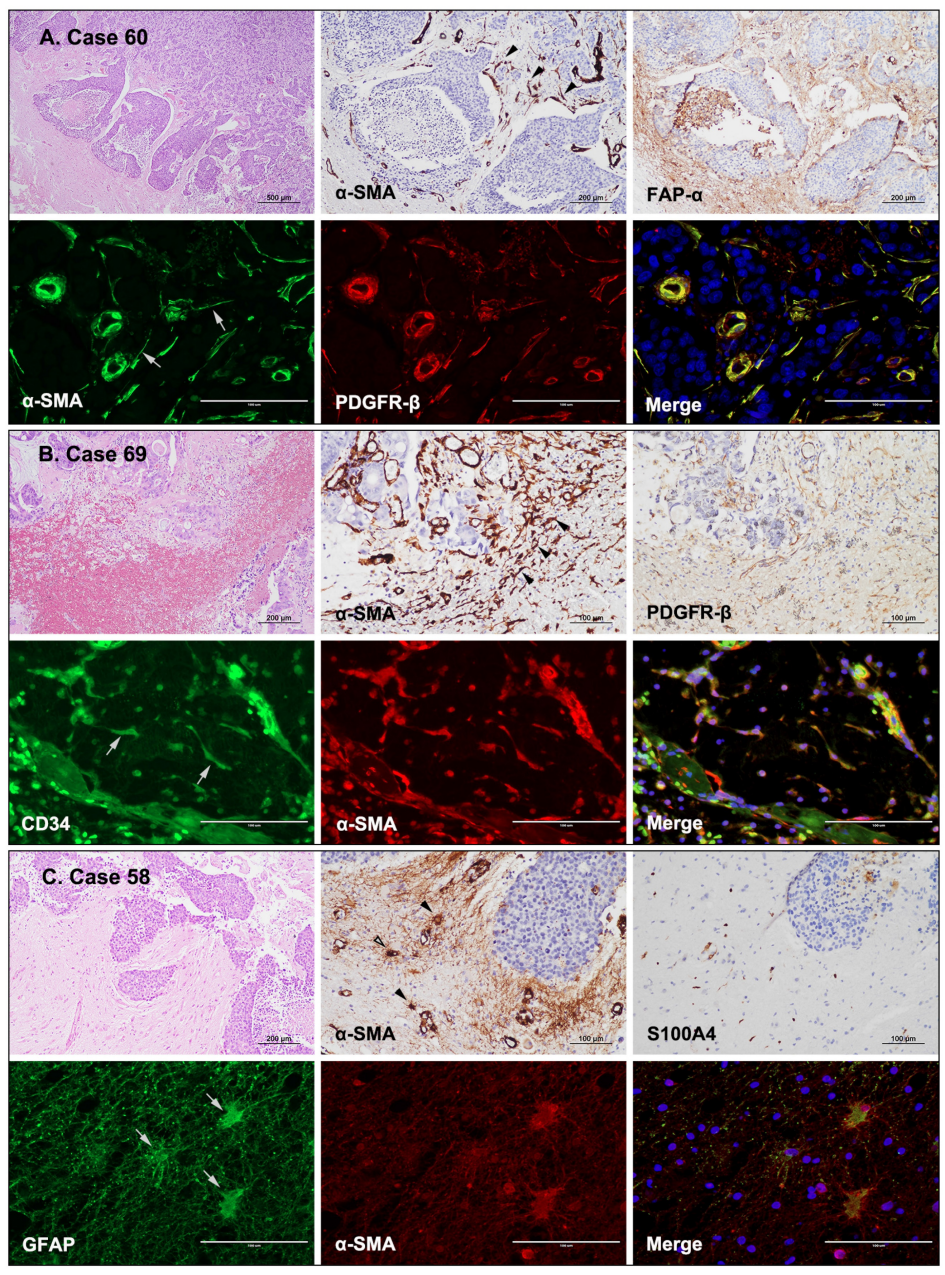
** Possible origin of CAFs in BMs based on the expression of CAF-related markers.** (a) A BM from a patient with breast cancer (case 60). The spindle-shaped cells surrounding the tumor nests expressed α-SMA IHC (black arrowheads) and were immunopositive for FAP-α. IF images showing co-expression of α-SMA (gray arrows) and PDGFR-β in CAFs that were connected to endothelial cells or pericytes of blood vessels (H&E stains, ×40; IHC, ×200; IF ×400). (b) A BM from a patient with colon cancer (case 69). The spindle-shaped cells in the hemorrhagic foci expressed α-SMA (black arrowheads) and PDGFR-β (IHC). IF revealed co-expression of CD34 (gray arrows) and α-SMA in these spindle-shaped cells, suggesting that they likely originated from endothelial progenitor cells or hematopoietic stem cells from the bone marrow (H&E stains, ×100; IHC, ×200; IF ×400). (c) A BM from a patient with breast cancer (case 58). Glial stroma in the peritumoral areas contained α-SMA-positive stellate cells (black arrowheads) with weak S100A4 expression. The stellate cells co-expressed GFAP (gray arrows) and α-SMA, suggesting that the CAFs might have developed from transformed astrocytes of the peritumoral glial stroma (H&E stains, ×100; IHC, ×200; IF ×400).

CAFs are often derived from mesoderm precursor cells; however, the exact origin of CAFs in the TME remains mostly unknown and is likely to be miscellaneous 9. In the present study, human FFPE BM tissues were examined using IHC to identify potential sources of stromal CAFs. CAFs appeared to be connected to endothelial cells or pericytes of blood vessels when α-SMA and FAP-α were expressed in the cells extending from the vessels (Fig. 3A). Moreover, stellate-shaped CAFs were present in the hemorrhagic spots within the tumor (Fig. 3B). In these cases, CAFs could have derived from endothelial progenitor cells or hematopoietic stem cells of the bone marrow that migrated to the tumor via the circulation. In addition, α-SMA and S100A4/FSP1 were co-expressed in astrocytes found in the glial stromal of peritumoral areas (Fig. 3C). Therefore, these CAFs could have developed from transformed astrocytes. IF staining revealed concurrent α-SMA and PDGFR-β expression in endothelial cells and pericytes of vessels. Interestingly, stellate-shaped cells extending from the periphery of the blood vessels also co-expressed α-SMA and PDGFR-β (Fig. 3A). Stellate-shaped cells located in peritumoral hemorrhagic sites co-expressed CD34 and α-SMA (Fig. 3B). In addition, the glial stroma in peritumoral areas contained astrocytes with GFAP and α-SMA co-expression (Fig. 3C). Taken together, these findings indicate that the observed stellate cells were presumed to be in transition states and that CAFs could originate from pericytes/endothelial cells of blood vessels, circulating endothelial progenitor cells/hematopoietic stem cells delivered, or transformed astrocytes of the peritumoral glial stroma.

### Relationship between clinicopathological characteristics and expression of CAF-related biomarkers with clinical relevance

We also analyzed the relationship between the clinicopathological variables in BM patients, including expression of CAF-related biomarkers, tumor size (large tumor size was defined as ≥3 cm in diameter), and local recurrence after surgical resection. From all CAF-related biomarkers assessed, univariate analyses showed that PDGFR-β, α-SMA, and collagen type I were significantly associated with BM size (*P* < 0.001, *P* = 0.002, and *P* = 0.023, respectively). Multivariate analyses revealed that high expression of PDGFR-β was the most potent predictor of BM size (hazard ratio [HR], 0.069; 95% confidence interval [CI], 0.017-0.284; *P* < 0.001) (Table 1). By contrast, S100A4/FSP1, FAP-α, and Twist1 were not significantly associated with BM size (*P* > 0.05, all). When chemotherapy or radiotherapy was performed for primary cancer prior to BM resection, the frequency of large BM sizes tended to increase, but there was no statistical significance (*P* > 0.05).

**Fig 4:**
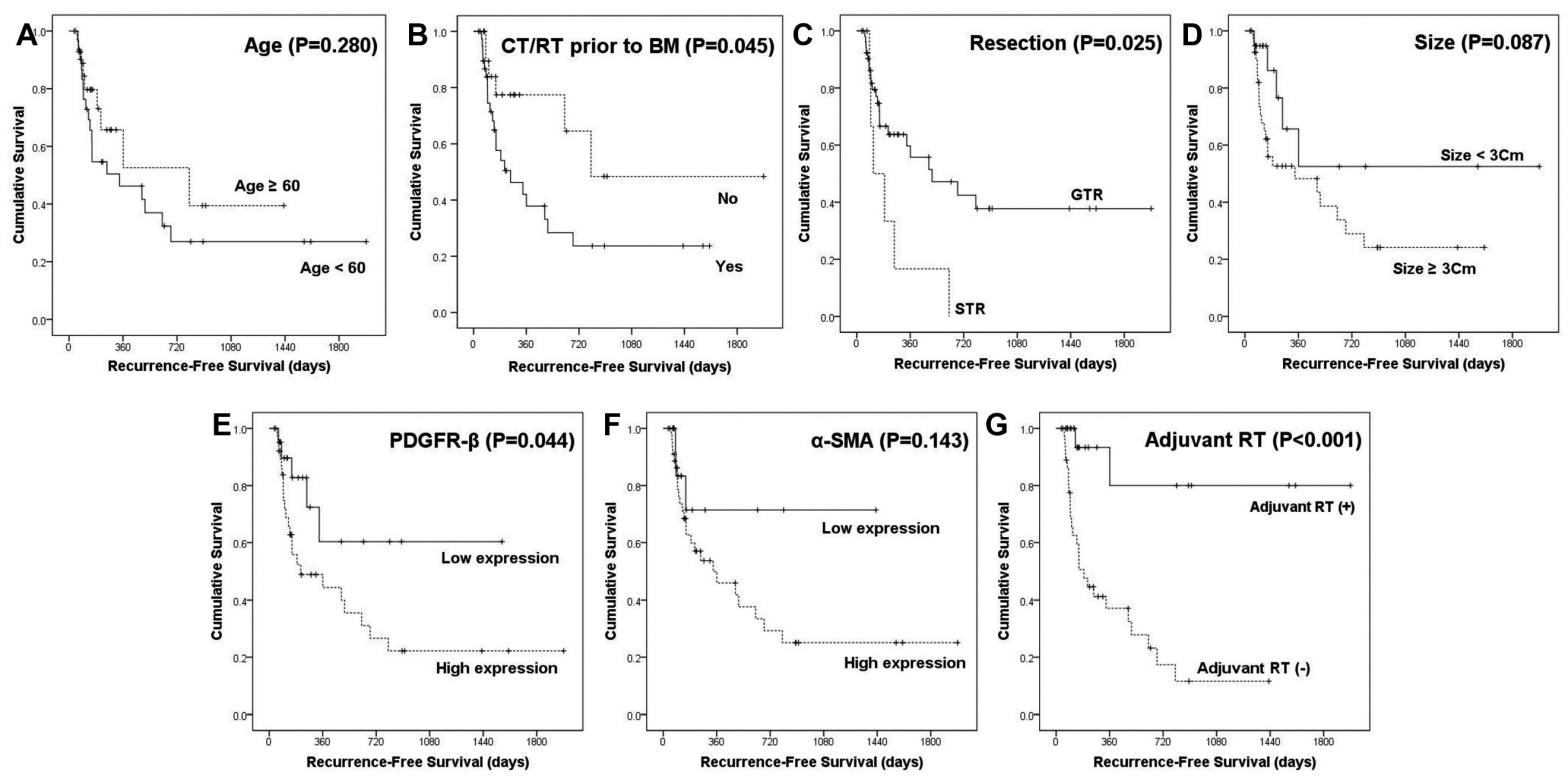
** Recurrence-free survival analyses using the Kaplan-Meier estimator and log-rank test.** The impact of different clinicopathological variables (age, chemotherapy or radiation therapy prior to brain metastasis, resection, size, and adjuvant radiation therapy) and expression of PDGFR-β and α-SMA on RFS were assessed.

Local recurrence after surgical resection was significantly associated with various factors, including age (*P* = 0.027), chemotherapy or radiotherapy for primary cancer prior to BM (*P* = 0.028), BM size (*P* = 0.039), adjuvant radiotherapy (RT; *P* < 0.001), PDGFR-β expression (*P* = 0.003), and α-SMA expression (*P* = 0.011). Among them, no adjuvant RT (HR, 0.028; 95% CI, 0.005-0.168; *P* < 0.001) and high expression of α-SMA (HR, 0.116; 95% CI, 0.02-0.673; *P* = 0.016) were independent predictors of BM local recurrence in patients with BM (Table 2). These results were supported by survival analyses using the Kaplan-Meier method (Fig. 4). Chemotherapy or radiotherapy for primary cancer prior to BM (*P* = 0.045), subtotal resection (*P* = 0.025), high expression of PDGFR-β (*P* = 0.044), and no adjuvant RT (*P* < 0.001) were significantly associated with shorter RFS. Furthermore, high expression of α-SMA was associated with worse RFS, yet this association did not reach statistical significance (*P* = 0.143).

In the analysis of the relationship between expression of CAF-related biomarkers and clinical variables, chemotherapy or radiotherapy for primary cancer prior to BM was significantly associated with high expression of PDGFR-β (*P* = 0.010), α-SMA (*P* = 0.002), and FSP1/S100A4 (*P* = 0.012) and marginally associated with Collagen type 1 and FAP-α (*P* = 0.073 and *P* = 0.080, respectively, Table 3).

### Isolation and characterization of primary CAFs and NFs

**Fig 5:**
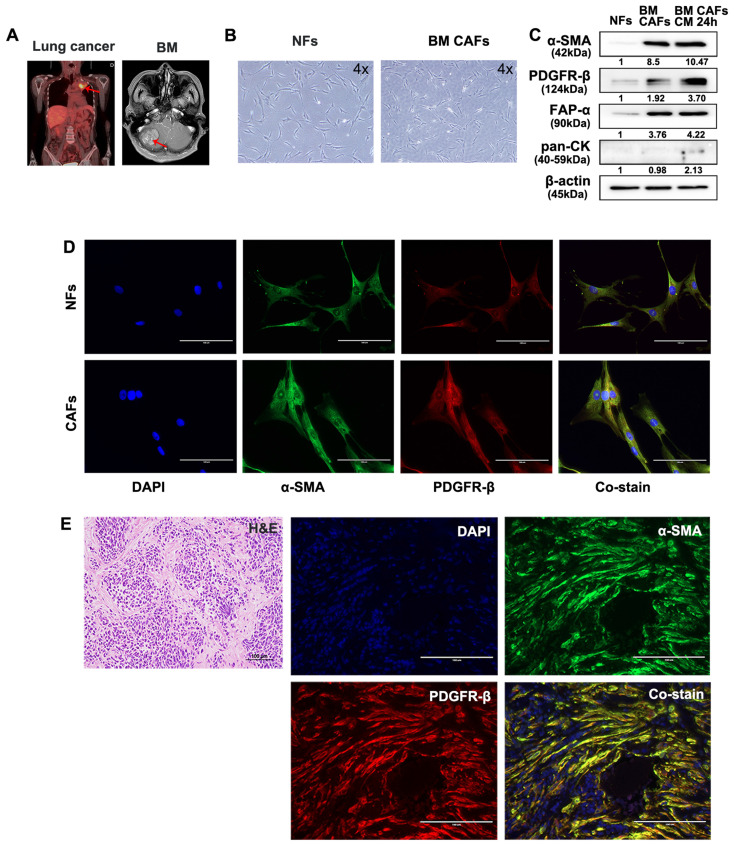
** Molecular characterization of patient-derived BM-CAFs and NFs from metastatic lung cancer.** (a) Magnetic resonance imaging of a 56-year-old female patient diagnosed with a BM from primary lung cancer. (b) Light microscopy images of NFs and CAFs isolated from fresh tissues (magnification, ×4). (c) Western blotting analyses revealed higher protein levels of α-SMA, PDGFR-β, and FAP-α in BM-CAFs and BM-CAFs cultured in conditioned media compared to primary NFs. All three cell types were negative for pan-cytokeratin (pan-CK). (d) IF staining indicated high levels of α-SMA and PDGFR-β in primary CAFs compared to primary NFs (IF, ×400). (e) IF staining on FFPE BM tissues showed high co-expression of α-SMA and PDGFR-β in peritumoral spindle-shaped cells, while tumor cells did not express these markers (IF, ×400).

**Fig 6:**
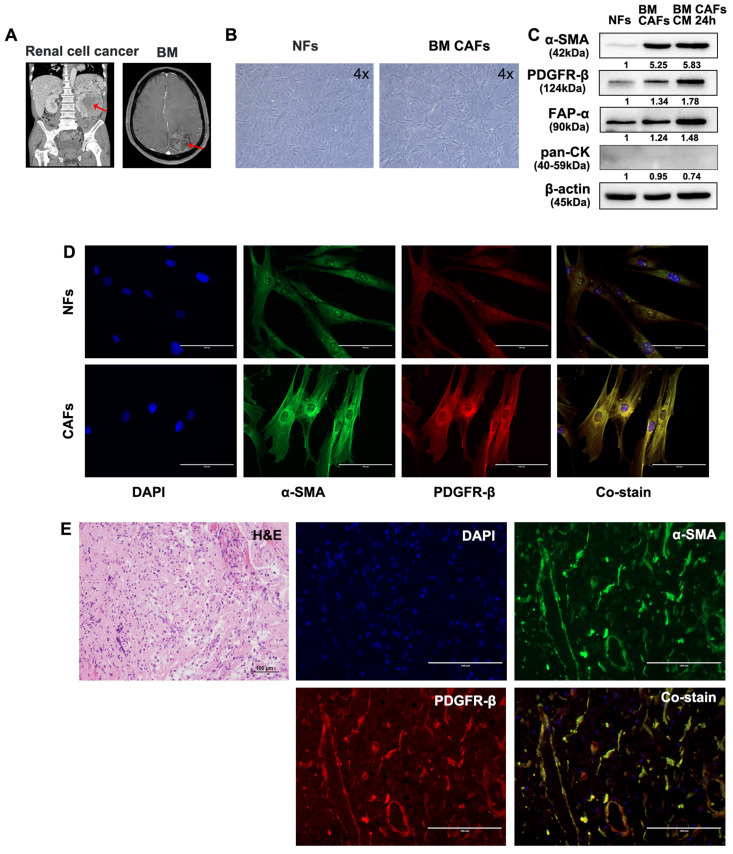
** Molecular characterization of patient-derived BM-CAFs and NFs from metastatic renal cell carcinoma.** (a) Magnetic resonance imaging of a 55-year-old male diagnosed with a BM from renal cell carcinoma. (b) Light microscopy images of NFs and CAFs isolated from fresh tissues (magnification, ×4). (c) Western blotting analyses revealed a higher expression level of α-SMA, PDGFR-β, and FAP-α in BM-CAFs and BM-CAFs treated with conditioned media compared to primary NFs. All cells were negative for pan-cytokeratin (pan-CK). (d) IF staining indicated high levels of α-SMA and PDGFR-β expression in primary CAFs compared to primary NFs (IF, ×400). (e) IF staining of FFPE BM tissues showed co-expression of α-SMA and PDGFR-β in peritumoral spindle-shaped cells (IF, ×400).

Fresh tumor and skin tissues from patients who underwent surgical resection of BM were used for CAF isolation and characterization. Samples from two patients with BMs from lung cancer and renal cell carcinoma were used for these analyses (Fig. 5A and 6A). Patient-derived BM-CAFs appeared to have a flattened, cruciform, and elongated morphology compared to the uniformly spindle-shaped NFs (Fig. 5B and 6B). Western blotting analyses revealed that the expression of CAF-related biomarkers, including α-SMA, PDGFR-β, and FAP-α, was much higher in BM-CAFs cultured in conditioned media for 24 h than in paired NFs and even higher than in BM-CAF cultured in conventional media, while pan-CK and epithelial markers were not expressed in BM-CAFs (Fig. 5C and 6C). In contrast to the usual high expression of pan-CK in A549 and MDA-MB-231, the fact that pan-CK was not expressed in BM CAFs cultured in conditioned media allows us to assume that conditioned media contains secreted substances that enhance the BM-CAF phenotype even without carcinoma cells. IF analyses confirmed the considerably higher expression of α-SMA and PDGFR-β in primary BM-CAFs compared to NFs (Fig. 5D and 6D). Dual IF staining performed on FFPE tissue from the same patients also indicated that α-SMA and PDGFR-β were co-localized in stromal BM-CAFs (Fig. 5E and 6E). By contrast, the expression of α-SMA and PDGFR-β was undetectable in various cancer cell lines, including LLC1, MDA-MB-231, A549, MC38, and GL261 (data not shown).

## Discussion

BM is a frequent event in advance-stage cancer patients and one of the leading causes of morbidity and mortality. Stromal CAFs have been implicated in carcinogenesis, metastasis, immune suppression, and drug resistance through the reciprocal interaction of cancer cells and CAFs in TME 25, 26. However, heterogeneity and lack of unique CAF markers remain significant challenges in the identification of therapeutic targets for cancer immunotherapy 27. In the present study, we investigated the expression of various CAF-related biomarkers in BM and patient-derived primary CAFs and NFs, and assessed their clinical significance. Our results suggest that PDGFR-β and α-SMA are the most clinically relevant CAF-related biomarkers in BM.

Fibroblast-related biomarkers exhibit distinct expression patterns in stromal CAFs in the TME of various tumor types 28, 29. In this study, we found that several CAF-related markers, including PDGFR-β, α-SMA, FAP-α, S100A4/FSP1, collagen type I, and Twist1, were expressed at high levels in stromal CAFs in the proximity of tumor areas in BMs. By contrast, NG2 and Tenascin-C were expressed at low levels, and only in a small number of BM cases. Among the investigated CAF-related biomarkers, PDGFR-β, α-SMA, and collagen type I were associated with a large BM size. Moreover, PDGFR-β and α-SMA were significantly associated with BM recurrence after resection. PDGFR-β in stromal cells has been shown to enhance EMT, cancer cell stemness, and angiogenesis, thereby promoting metastasis and resulting in poor survival in patients with prostate cancer 30 and in patients with BM of breast cancer 31. An active PDGF domain is capable of inducing PDGF-β receptor phosphorylation, promoting cell proliferation and migration in an autocrine mechanism 32. α-SMA is a promising biomarker of metastasis, therapeutic resistance, and adverse clinical prognosis, and has been linked to EMT and epithelial stemness in lung and kidney cancers 33, 34. Consistent with our findings, abundant expression of stromal α-SMA in colon and breast cancers is associated with poor prognosis 35, 36. Other biomarkers investigated in this study are important players in TME modulation. FAP-α regulates ECM remodeling, as well as cancer cell invasion and migration 37, while S100A4/FSP1 is involved in angiogenesis, invasion, and metastatic colonization 38. Twist1 is an EMT marker that is expressed in CAFs found in the TME. CAFs expressing Twist1 were shown to originate from malignant epithelial cells and to have potent EMT-promoting functions 21. A double-edged role of collagen has been reported in cancer fibrosis 39. Recent investigation revealed that the secretion and alignment of collagen type I was regulated by subpopulation of CAF 40. Furthermore, NG2 has been demonstrated to enhance tumor cell invasion and metastasis through β-1 integrin activation 41, while Tenascin-C has been linked to tumor invasiveness and metastatic progression 42. The characteristics of the used CAF markers are summarized in Table 4.

Our findings suggest that CAF-related biomarkers are associated with BM progression and poor clinical outcomes. Age (<60 years), chemotherapy or radiotherapy for primary cancer prior to BM, a large BM size (≥3 cm in diameter), subtotal resection (STR) and no adjuvant radiation therapy were correlated with BM recurrence after surgical resection. Previous clinical studies have reported similar results regarding the relationship between BM prognosis and the primary cancer types they originated from 43-45. The patient population selected in the present study consisted of small patient groups with different primary cancers; thus, we could not compare the prognosis between groups of patients with different primary cancers, due to the insufficient number of patients. In addition, we found that chemotherapy or radiotherapy for primary cancer prior to BM resection was significantly associated with higher frequency of local recurrence (56.4%) compared to the group without chemotherapy or radiotherapy (27.3%). Also, the size of BM tended to be larger in the group with chemotherapy or radiotherapy prior to BM without statistical significance. Pre-operative chemotherapy or radiotherapy prior to BM may have affected the promotion of metastasis, but limited clinical trials have been performed. Furthermore, the effects of radiation therapy on metastasis remain still controversial. Previous studies revealed that chemotherapy for the primary tumor induced chemo-resistance and facilitated metastatic colonization, recruitment of immune and stromal cells and tumor recurrence through angiogenesis as well 46, 47. We also observed that high expression of CAF markers (PDGFR-β, α-SMA and FSP1) was significantly linked to the presence of chemotherapy or radiotherapy for systemic cancer prior to BM resection. Previous studies suggested that α-SMA-positive CAFs became enriched into rectal cancer following neoadjuvant therapy and were correlated with poor prognosis represented by shortened recurrence-free survival 48, 49. Chemotherapy or radiotherapy has been known to affect CAFs through activating TME networks by triggering immune reactions, vascularization, fibrosis and, in particular, by secreting cytokines, chemokines and growth factors 49.

Although the origins of CAFs are not fully understood, they may originate from resident fibroblasts 50, pericytes or vascular smooth muscle cells 51, epithelial cells, endothelial cells 5, and bone marrow-derived mesenchymal stem cells 52. IF staining of BM tissues revealed α-SMA and PDGFR-β co-expression in pericytes or endothelial cells in blood vessels. Hemorrhagic endothelial progenitor cells from the bone marrow co-expressed CD34 and α-SMA, while peritumoral astrocytes co-expressed GFAP and α-SMA. All of these cells might contribute significantly to the formation of a peritumoral “reactive stroma,” as they have several myofibroblast-specific features and the ability to produce a variety of pro-invasive molecules 53, 54. For instance, recent studies have demonstrated that CAFs that originate from myofibroblasts, pericytes, and mesenchymal stem cells are important regulators of cancer initiation, growth, progression, and metastasis 55-57.

In this study, we found significant differences between primary CAFs and NFs based on morphologic assessments and molecular analyses. The expression of α-SMA and PDGFR-β was elevated in CAFs compared to NFs, suggesting that fibroblasts may undergo partial phenotypic changes during tumor progression and metastasis. Earlier studies have reported that PDGFR-β and α-SMA expression is significantly higher in CAFs than in NFs 58-60. Pan-CK, a representative epithelial cell marker, was not expressed in primary CAFs or NFs, confirming their distinction from parenchymal cancer cells. Exposure to conditioned media induced the expression of PDGFR-β and α-SMA in BM CAFs; hence, the phenotypic changes in CAFs could be regulated by metabolites and growth factors found in conditioned media of cancer cells 21. High expression levels of PDGFR-β and α-SMA in stromal CAFs compared to the tumor cells supported the clear difference between CAFs and parenchymal cancer cells, similarly to what previous studies have reported 30, 61. Considering the distinct expression patterns of CAF-related markers and the varying distribution of CAFs in BMs from different primary cancer types, the unique characteristics of the tumor stroma are likely formed through reciprocal and paracrine interactions between cancer cells and CAFs.

The existence of CAFs in BM has not been clearly established, and much less their role has been studied. The current study explored the presence and distribution of BM CAFs and contains unprecedented trials of primary isolation and culture of BM CAFs from patient-derived tissue samples. Although limited by the insufficient number of the BM cases and the diversity of primary cancers, we showed that the expression of specific BM-CAF markers, such as α-SMA and PDGFR-β, is associated with clinical characteristics and prognosis. We also described the distribution pattern of BM CAFs and their potential cell origin through double fluorescence staining. It will provide the basis for further studies on the clinical significance of these factors in the future.

Still there are many limitations in this study. This retrospective study is highly likely to have a selection bias by including various primary organ cancers while selecting a relatively small number of patients. In addition, our study was conducted mainly using IHC in a single institutional patient sample, and an external validation method could be carried out for more objective analysis. In order to increase the reliability of the results of this study, large cohort studies from multicenter are desirable. Nonetheless, this study is the first to comprehensively analyze the relationship between CAF features, expression of CAFs-related biomarkers, clinicopathological characteristics, and patient outcomes in BMs from various primary cancer types. Nevertheless, further genetic analyses, comprehensive immune profiling, and animal studies need to be performed for a better understanding of the clinical relevance of CAF-specific biomarkers and the role and origins of CAFs in the TME.

In conclusion, PDGFR-β and α-SMA are robust CAF biomarkers in BM, strongly associated with poor clinical outcomes. The elucidation of the role of CAFs in BM progression will enable a better understanding of the TME and contribute to strategic treatment of BMs.

## Supplementary Material

Supplementary figure and table.

## Figures and Tables

**Table 1:** Association between BM size and clinicopathological characteristics or expression of CAF biomarkers.

Variables		Large BM (size ≥ 3cm)	Univariate	Multivariate		
			*P*-value	HR	95% CI	*P*-value
General characteristics						
Age (yrs)	< 60	68.8% (22/32)	0.986			0.555
	≥ 60	69.0% (20/29)				
Sex	Female	75.0% (18/24)	0.404			0.720
	Male	64.9% (24/37)				
CT or RT prior to BM	Absent	59.1% (13/22)	0.216			0.623
	Present	74.4% (29/39)				
Other metastasis^!^	Absent	65.6% (21/32)	0.357			0.193
	Present	77.3% (17/22)				
KPS	≥ 90	71.7% (33/46)	0.394			0.732
	< 90	60.0% (9/15)				
Symptoms	None to minimal	72.4% (21/29)	0.567			0.890
	Moderate to severe	65.6% (21/32)				
BM characteristics						
Detection time of BM	Metachronous	72.9% (35/48)	0.188			NA^*^
	Synchronous	53.8% (7/13)				
Number of BM	Single	64.3% (27/42)	0.252			0.291
	Multiple	78.9% (15/19)				
Location	Supratentotial	64.7% (33/51)	0.114			0.380
	Infratentorial	90.0% (9/10)				
Resection	GTR	68.5% (37/54)	0.876			NA^#^
	non-GTR	71.4% (5/7)				
Adjuvant RT	Yes	56.0% (14/25)	0.071			NA^#^
	No	77.8% (28/36)				
Recurrence	No	57.6% (19/33)	0.039			NA^#^
	Yes	82.1% (23/28)				
CAF biomarkers expression						
PDGFR-β	Low	39.1% (9/23)	<0.001	0.069	0.017-0.284	<0.001
	High	86.8% (33/38)				
α-SMA	Low	37.5% (6/16)	0.002			0.587
	High	80.0% (36/45)				
Collagen I	Low	41.5% (5/12)	0.023			0.698
	High	75.5% (37/49)				
FSP1/S100A4	Low	64.3% (9/14)	0.674			0.614
	High	70.2% (33/47)				
FAP-α	Low	66.7% (18/27)	0.743			0.595
	High	70.6% (24/34)				
Twist1	Low	62.5% (20/32)	0.260			0.804
	High	75.9% (22/29)				

CAF, cancer-associated fibroblast; CT, chemotherapy; RT, radiation therapy; BM, brain metastasis; HR, hazard ratio; CI, confidence interval; KPS, Karnofsky performance score; GTR, gross total resection; NA^*^, excluded in multivariate analysis due to collinearity with CT or RT prior to BM; NA^#^, excluded in multivariate analysis because they were postoperative parameters; !, in 54 patients performed systemic evaluation of cancer status at BM detection.

**Table 2:** Association between BM recurrence after resection and clinicopathological characteristics or CAF marker expression.

Variables		Frequency of recurrence	Univariate	Multivariate		
			*P*-value	HR	95% CI	*P*-value
General characteristics						
Age (yrs)	< 60	59.4% (19/32)	0.027			0.398
	≥ 60	31.0% (9/29)				
Sex	Female	54.2% (13/24)	0.297			0.502
	Male	40.5% (15/37)				
CT or RT prior to BM	Absent	27.3% (6/22)	0.028			0.425
	Present	56.4% (22/39)				
Other metastasis^#^	Absent	53.1% (17/32)	0.821			0.582
	Present	50.0% (11/22)				
KPS	≥ 90	47.8% (22/46)	0.597			0.791
	< 90	40.0% (6/15)				
Symptoms	None to minimal	51.7% (15/29)	0.385			0.648
	Moderate to severe	40.6% (13/32)				
BM characteristics						
Detection time of BM	Metachronous	50.0% (24/48)	0.217			NA^*^
	Synchronous	30.8% (4/13)				
Number of BM	Single	42.9% (18/42)	0.478			0.891
	Multiple	52.6% (10/19)				
Size in diameter	< 3cm	26.3% (5/19)	0.039			0.998
	≥ 3cm	54.8% (23/42)				
Location	Supratentotial	43.1% (22/51)	0.328			0.471
	Infratentorial	60.0% (6/10)				
Resection	GTR	40.7% (22/54)	0.025			0.330
	non-GTR	85.7% (6/7)				
Adjuvant RT	Yes	8.0% (2/25)	<0.001	0.028	0.005-0.168	<0.001
	No	72.2% (26/36)				
CAF markers expression						
PDGFR-β	Low	21.7% (5/23)	0.003			0.651
	High	60.5% (23/38)				
α-SMA	Low	18.8% (3/16)	0.011	0.116	0.020-0.673	0.016
	High	55.6% (25/45)				
Collagen I	Low	33.3% (4/12)	0.330			0.730
	High	49.0% (24/49)				
FSP1/S100A4	Low	42.9% (6/14)	0.795			0.607
	High	46.8% (22/47)				
FAP-α	Low	48.1% (13/27)	0.754			0.519
	High	44.1% (15/34)				
Twist1	Low	37.5% (12/32)	0.167			0.732
	High	55.2% (16/29)				

CAF, cancer-associated fibroblast; CT, chemotherapy; RT, radiation therapy; BM, brain metastasis; HR, hazard ratio; CI, confidence interval; KPS, Karnofsky performance score; GTR, gross total resection; NA^*^, excluded in multivariate analysis due to collinearity with CT or RT prior to BM; #, in 54 patients performed systemic evaluation of cancer status at BM detection.

**Table 3:** Association between clinical characteristics and CAF marker expression.

Variables		High PDGFR-β	High α-SMA	High Collagen 1	High FSP1/S100A4	High FAP-α	High Twist1
Age (yrs)	< 60	62.5%	81.3%	81.3%	75.0%	62.5%	53.1%
	≥ 60	62.1%	65.5%	79.2%	79.3%	48.3%	41.4%
	*P*-value	0.972	0.163	0.849	0.689	0.264	0.359
Sex	Female	75.0%	75.0%	79.2%	75.0%	70.8%	45.8%
	Male	54.1%	73.0%	81.1%	78.4%	45.9%	48.6%
	*P*-value	0.099	0.860	0.854	0.759	0.056	0.830
CT or RT prior to BM	Absent	40.9%	50.0%	68.2%	59.1%	40.9%	36.4%
	Present	74.4%	87.2%	87.2%	87.2%	64.1%	53.8%
	*P*-value	0.010	0.002	0.073	0.012	0.080	0.189
Other metastasis	Absent	71.9%	78.1%	71.9%	78.1%	53.0%	56.3%
	Present	68.2%	68.2%	86.4%	72.7%	59.1%	40.9%
	*P*-value	0.770	0.413	0.208	0.648	0.665	0.268
Symptoms	None to minimal	72.4%	79.3%	86.2%	89.7%	55.2%	51.7%
	Moderate to severe	53.1%	68.8%	75.0%	65.6%	56.3%	43.8%
	*P*-value	0.121	0.349	0.272	0.026	0.933	0.533
Detection time of BM	Metachronous	68.8%	81.3%	85.4%	83.3%	60.4%	50.0%
	Synchronous	38.5%	46.2%	61.5%	53.8%	38.5%	38.5%
	*P*-value	0.046	0.011	0.055	0.025	0.157	0.460
Number of BM	Single	61.9%	69.0%	76.2%	76.2%	54.8%	45.2%
	Multiple	63.2%	84.2%	89.5%	78.9%	57.9%	52.6%
	*P*-value	0.925	0.212	0.227	0.813	0.820	0.592
Location	Supratentotial	56.9%	76.5%	82.4%	76.5%	52.9%	47.1%
	Infratentorial	90.0%	60.0%	70.0%	80.0%	70.0%	50.0%
	*P*-value	0.048	0.279	0.369	0.808	0.321	0.865

CAF, cancer-associated fibroblast; CT, chemotherapy; BM, brain metastasis; GTR, gross total resection; RT, radiation therapy. Chi-square test was used for univariate analysis.

**Table 4:** Summary of markers for identification of CAFs

Potential CAF markers	Biochemical and molecular characteristics
α-SMA (alpha-smooth muscle actin)	Important role in cell motility, structure and integrity 29. prognostic significance in breast and colon cancer 35, 36.
FAP-α (fibroblast activation protein α)	Type II integral membrane protein from the membrane-bound serine protease family 29. Regulation of epithelial-mesenchymal transition (EMT) 37.
S100A4 (S100 calcium binding protein A4) /FSP1 (fibroblast-specific protein 1)	Involved in angiogenesis, invasion, and metastatic colonization 38. Common fibroblast marker but considered to be a marker for quiescent fibroblasts rather than CAFs 29.
PDGFR (platelet-derived growth factor receptor)-α	Widely expressed over the larger fibroblast population. Mutated in approximately 10% of gastrointestinal stromal tumor patients 29.
PDGFR-β	Tyrosine kinase receptors on the surface of fibroblasts, astrocytes, neuroprogenitors and pericytes 29. Enhance EMT, cancer cell stemness, and angiogenesis 30.
Collagen type I	Expressed in myofibroblast subtype 40.
NG2 (nerve glial antigen 2)	Enhance tumor cell invasion and metastasis through integrin activation 41. Also positive in by numerous other cells, such as myeloid and T-cells 27.
Tenascin-C	Member of the extracellular matrix. Linked to tumor invasiveness and metastatic progression 20.
Twist1	Basic helix-loop-helix transcription factor. Important inducer of EMT 21.

## Abbreviations

α-SMA: Alpha-smooth muscle actin
BM: Brain metastasis
CAF: Cancer-associated fibroblast
IHC: Immunohistochemistry
IF: Immunofluorescence
PDGFR-β: Platelet-derived growth factor receptor
TME: Tumor microenvironment

## References

[1] Achrol AS, Rennert RC, Anders C, Soffietti R, Ahluwalia MS, Nayak L (2019). Brain metastases. Nat Rev Dis Primers.

[2] Arvold ND, Lee EQ, Mehta MP, Margolin K, Alexander BM, Lin NU (2016). Updates in the management of brain metastases. Neuro Oncol.

[3] Brastianos HC, Cahill DP, Brastianos PK (2015). Systemic therapy of brain metastases. Curr Neurol Neurosci Rep.

[4] Lambert AW, Pattabiraman DR, Weinberg RA (2017). Emerging Biological Principles of Metastasis. Cell.

[5] Bu L, Baba H, Yoshida N, Miyake K, Yasuda T, Uchihara T (2019). Biological heterogeneity and versatility of cancer-associated fibroblasts in the tumor microenvironment. Oncogene.

[6] Pereira BA, Vennin C, Papanicolaou M, Chambers CR, Herrmann D, Morton JP (2019). CAF Subpopulations: A New Reservoir of Stromal Targets in Pancreatic Cancer. Trends Cancer.

[7] Monteran L, Erez N (2019). The Dark Side of Fibroblasts: Cancer-Associated Fibroblasts as Mediators of Immunosuppression in the Tumor Microenvironment. Front Immunol.

[8] Fiori ME, Di Franco S, Villanova L, Bianca P, Stassi G, De Maria R (2019). Cancer-associated fibroblasts as abettors of tumor progression at the crossroads of EMT and therapy resistance. Mol Cancer.

[9] LeBleu VS, Kalluri R (2018). A peek into cancer-associated fibroblasts: origins, functions and translational impact. Dis Model Mech.

[10] Paulsson J, Micke P (2014). Prognostic relevance of cancer-associated fibroblasts in human cancer. Semin Cancer Biol.

[11] Gieniec KA, Butler LM, Worthley DL, Woods SL (2019). Cancer-associated fibroblasts-heroes or villains?. Br J Cancer.

[12] Ohlund D, Handly-Santana A, Biffi G, Elyada E, Almeida AS, Ponz-Sarvise M (2017). Distinct populations of inflammatory fibroblasts and myofibroblasts in pancreatic cancer. J Exp Med.

[13] Herrera M, Islam AB, Herrera A, Martin P, Garcia V, Silva J (2013). Functional heterogeneity of cancer-associated fibroblasts from human colon tumors shows specific prognostic gene expression signature. Clin Cancer Res.

[14] Zeine R, Salwen HR, Peddinti R, Tian Y, Guerrero L, Yang Q (2009). Presence of cancer-associated fibroblasts inversely correlates with Schwannian stroma in neuroblastoma tumors. Mod Pathol.

[15] Li YY, Tao YW, Gao S, Li P, Zheng JM, Zhang SE (2018). Cancer-associated fibroblasts contribute to oral cancer cells proliferation and metastasis via exosome-mediated paracrine miR-34a-5p. EBioMedicine.

[16] Sandberg TP, Stuart M, Oosting J, Tollenaar R, Sier CFM, Mesker WE (2019). Increased expression of cancer-associated fibroblast markers at the invasive front and its association with tumor-stroma ratio in colorectal cancer. BMC Cancer.

[17] Primac I, Maquoi E, Blacher S, Heljasvaara R, Van Deun J, Smeland HY (2019). Stromal integrin alpha11 regulates PDGFR-beta signaling and promotes breast cancer progression. J Clin Invest.

[18] Garcia-Pravia C, Galvan JA, Gutierrez-Corral N, Solar-Garcia L, Garcia-Perez E, Garcia-Ocana M (2013). Overexpression of COL11A1 by cancer-associated fibroblasts: clinical relevance of a stromal marker in pancreatic cancer. PLoS One.

[19] Marsh T, Pietras K, McAllister SS (2013). Fibroblasts as architects of cancer pathogenesis. Biochim Biophys Acta.

[20] Ni WD, Yang ZT, Cui CA, Cui Y, Fang LY, Xuan YH (2017). Tenascin-C is a potential cancer-associated fibroblasts marker and predicts poor prognosis in prostate cancer. Biochem Biophys Res Commun.

[21] Lee KW, Yeo SY, Sung CO, Kim SH (2015). Twist1 is a key regulator of cancer-associated fibroblasts. Cancer Res.

[22] Cortez E, Roswall P, Pietras K (2014). Functional subsets of mesenchymal cell types in the tumor microenvironment. Semin Cancer Biol.

[23] Noh MG, Oh SJ, Ahn EJ, Kim YJ, Jung TY, Jung S (2017). Prognostic significance of E-cadherin and N-cadherin expression in Gliomas. BMC Cancer.

[24] Lee KH, Ahn EJ, Oh SJ, Kim O, Joo YE, Bae JA (2015). KITENIN promotes glioma invasiveness and progression, associated with the induction of EMT and stemness markers. Oncotarget.

[25] Valkenburg KC, de Groot AE, Pienta KJ (2018). Targeting the tumour stroma to improve cancer therapy. Nat Rev Clin Oncol.

[26] Quail DF, Joyce JA (2013). Microenvironmental regulation of tumor progression and metastasis. Nat Med.

[27] Ohlund D, Elyada E, Tuveson D (2014). Fibroblast heterogeneity in the cancer wound. J Exp Med.

[28] Paauwe M, Schoonderwoerd MJA, Helderman R, Harryvan TJ, Groenewoud A, van Pelt GW (2018). Endoglin Expression on Cancer-Associated Fibroblasts Regulates Invasion and Stimulates Colorectal Cancer Metastasis. Clin Cancer Res.

[29] Nurmik M, Ullmann P, Rodriguez F, Haan S, Letellier E (2020). In search of definitions: Cancer-associated fibroblasts and their markers. Int J Cancer.

[30] Hagglof C, Hammarsten P, Josefsson A, Stattin P, Paulsson J, Bergh A (2010). Stromal PDGFRbeta expression in prostate tumors and non-malignant prostate tissue predicts prostate cancer survival. PLoS One.

[31] Thies KA, Hammer AM, Hildreth BE 3rd, Steck SA, Spehar JM, Kladney RD (2021). Stromal Platelet-Derived Growth Factor Receptor-beta Signaling Promotes Breast Cancer Metastasis in the Brain. Cancer Res.

[32] Heldin CH, Lennartsson J (2013). Structural and functional properties of platelet-derived growth factor and stem cell factor receptors. Cold Spring Harb Perspect Biol.

[33] Lee HW, Park YM, Lee SJ, Cho HJ, Kim DH, Lee JI (2013). Alpha-smooth muscle actin (ACTA2) is required for metastatic potential of human lung adenocarcinoma. Clin Cancer Res.

[34] Chen Q, Yang D, Zong H, Zhu L, Wang L, Wang X (2017). Growth-induced stress enhances epithelial-mesenchymal transition induced by IL-6 in clear cell renal cell carcinoma via the Akt/GSK-3beta/beta-catenin signaling pathway. Oncogenesis.

[35] Tsujino T, Seshimo I, Yamamoto H, Ngan CY, Ezumi K, Takemasa I (2007). Stromal myofibroblasts predict disease recurrence for colorectal cancer. Clin Cancer Res.

[36] Yamashita M, Ogawa T, Zhang X, Hanamura N, Kashikura Y, Takamura M (2012). Role of stromal myofibroblasts in invasive breast cancer: stromal expression of alpha-smooth muscle actin correlates with worse clinical outcome. Breast Cancer.

[37] Lee HO, Mullins SR, Franco-Barraza J, Valianou M, Cukierman E, Cheng JD (2011). FAP-overexpressing fibroblasts produce an extracellular matrix that enhances invasive velocity and directionality of pancreatic cancer cells. BMC Cancer.

[38] Boye K, Maelandsmo GM (2010). S100A4 and metastasis: a small actor playing many roles. Am J Pathol.

[39] Xu S, Xu H, Wang W, Li S, Li H, Li T (2019). The role of collagen in cancer: from bench to bedside. J Transl Med.

[40] Wu SZ, Roden DL, Wang C, Holliday H, Harvey K, Cazet AS (2020). Stromal cell diversity associated with immune evasion in human triple-negative breast cancer. Embo J.

[41] Stallcup WB (2017). NG2 Proteoglycan Enhances Brain Tumor Progression by Promoting Beta-1 Integrin Activation in both Cis and Trans Orientations. Cancers (Basel).

[42] Lowy CM, Oskarsson T (2015). Tenascin C in metastasis: A view from the invasive front. Cell Adh Migr.

[43] Maurer C, Tulpin L, Moreau M, Dumitrescu C, de Azambuja E, Paesmans M (2018). Risk factors for the development of brain metastases in patients with HER2-positive breast cancer. ESMO Open.

[44] Connolly EP, Mathew M, Tam M, King JV, Kunnakkat SD, Parker EC (2013). Involved field radiation therapy after surgical resection of solitary brain metastases-mature results. Neuro Oncol.

[45] Abel RJ, Ji L, Yu C, Lederman A, Chen T, Liu C (2015). Stereotactic radiosurgery to the resection cavity for brain metastases: prognostic factors and outcomes. J Radiosurg SBRT.

[46] D'Alterio C, Scala S, Sozzi G, Roz L, Bertolini G (2020). Paradoxical effects of chemotherapy on tumor relapse and metastasis promotion. Semin Cancer Biol.

[47] Barker HE, Paget JT, Khan AA, Harrington KJ (2015). The tumour microenvironment after radiotherapy: mechanisms of resistance and recurrence. Nat Rev Cancer.

[48] Verset L, Tommelein J, Moles Lopez X, Decaestecker C, Boterberg T, De Vlieghere E (2015). Impact of neoadjuvant therapy on cancer-associated fibroblasts in rectal cancer. Radiother Oncol.

[49] Wang Z, Tang Y, Tan Y, Wei Q, Yu W (2019). Cancer-associated fibroblasts in radiotherapy: challenges and new opportunities. Cell Commun Signal.

[50] Kobayashi H, Enomoto A, Woods SL, Burt AD, Takahashi M, Worthley DL (2019). Cancer-associated fibroblasts in gastrointestinal cancer. Nat Rev Gastroenterol Hepatol.

[51] Hosaka K, Yang Y, Seki T, Fischer C, Dubey O, Fredlund E (2016). Pericyte-fibroblast transition promotes tumor growth and metastasis. Proc Natl Acad Sci U S A.

[52] Kurashige M, Kohara M, Ohshima K, Tahara S, Hori Y, Nojima S (2018). Origin of cancer-associated fibroblasts and tumor-associated macrophages in humans after sex-mismatched bone marrow transplantation. Commun Biol.

[53] Zigrino P, Loffek S, Mauch C (2005). Tumor-stroma interactions: their role in the control of tumor cell invasion. Biochimie.

[54] De Wever O, Demetter P, Mareel M, Bracke M (2008). Stromal myofibroblasts are drivers of invasive cancer growth. Int J Cancer.

[55] Kalluri R, Zeisberg M (2006). Fibroblasts in cancer. Nat Rev Cancer.

[56] Chen YT, Chang FC, Wu CF, Chou YH, Hsu HL, Chiang WC (2011). Platelet-derived growth factor receptor signaling activates pericyte-myofibroblast transition in obstructive and post-ischemic kidney fibrosis. Kidney Int.

[57] Fregni G, Quinodoz M, Moller E, Vuille J, Galland S, Fusco C (2018). Reciprocal modulation of mesenchymal stem cells and tumor cells promotes lung cancer metastasis. EBioMedicine.

[58] Louault K, Bonneaud TL, Seveno C, Gomez-Bougie P, Nguyen F, Gautier F (2019). Interactions between cancer-associated fibroblasts and tumor cells promote MCL-1 dependency in estrogen receptor-positive breast cancers. Oncogene.

[59] Wei L, Ye H, Li G, Lu Y, Zhou Q, Zheng S (2018). Cancer-associated fibroblasts promote progression and gemcitabine resistance via the SDF-1/SATB-1 pathway in pancreatic cancer. Cell Death Dis.

[60] Sha M, Jeong S, Qiu BJ, Tong Y, Xia L, Xu N (2018). Isolation of cancer-associated fibroblasts and its promotion to the progression of intrahepatic cholangiocarcinoma. Cancer Med.

[61] Sugimoto H, Mundel TM, Kieran MW, Kalluri R (2006). Identification of fibroblast heterogeneity in the tumor microenvironment. Cancer Biol Ther.

