# Supplementary material for: Different Expression and Clinical Implications of Cancer-Associated Fibroblast (CAF) Markers in Brain Metastases
Source: J Cancer. 2023 Feb 5;14(3):464–79. doi: 10.7150/jca.80115 (PMC9969586; doi:10.7150/jca.80115)
Supplement: Supplementary file 1 — Supplementary figure and table. [file jcav14p0464s1.pdf]

Supplementary Materials:

Fig. S1. Weakly expressed CAF-related markers in BM-CAFs. (A) Twist1 was expressed in the nuclei of stromal CAFs of BM at variable levels according to primary cancer type. (B) NG2 and Tenascin-C were weakly expressed in a small number of cases, while PDGFR-α was expressed only in parenchymal tumor cells.

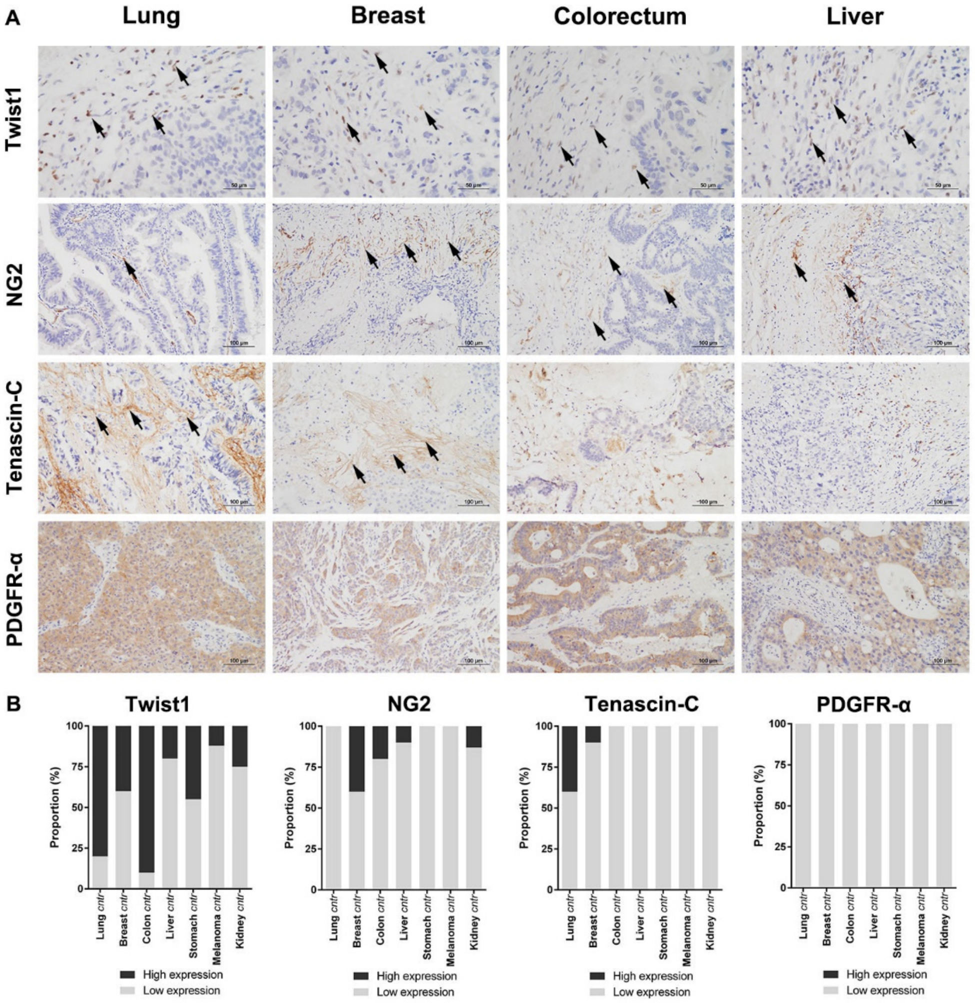

Supplementary Table 1. Case numbers of primary organ cancers at each selection step and the composition of histologic diagnoses

Primary organs	No. of cases			Primary diagnosis
	1st selection	2nd selection	Final selection	
Lung	228	192	10	6 ADC, 2SQC, 2 ASQC
Breast	50	47	10	9 IDC, 1 ILC
Colorectum	32	28	10	10 ADC
Liver	25	17	10	8 HCC, 2 CCA
Stomach	15	11	11	7 Intestinal, 3 Diffuse, 1NEC
Melanoma	11	9	9	5 Skin, 3 Unknown
Kidney	10	8	8	5 CRCC, 2 ChRCC, 1 NEC
Thyroid	11	3	0	
Total	382	315	68	

ADC, adenocarcinoma; SQC, squamous cell carcinoma; ASQC, adenosquamous carcinoma; IDC, invasive ductal carcinoma; ILC, invasive lobular carcinoma; HCC, hepatocellular carcinoma; CCA, cholangiocarcinoma; Intestinal, intestinal-type gastric adenocarcinoma; Diffuse, diffuse-type gastric adenocarcinoma; NEC, neuroendocrine carcinoma; Skin, melanoma of skin primary; Unknown, melanoma of unknown primary; CRCC, Clear cell renal cell carcinoma; ChRCC, Chromophobe renal cell carcinoma.
